# Thirteenth Annual ENBDC Workshop: Methods in Mammary Gland Biology and Breast Cancer

**DOI:** 10.1007/s10911-022-09526-6

**Published:** 2022-10-15

**Authors:** Alecia-Jane Twigger, Jakub Sumbal, Mohamed Bentires-Alj, Beatrice A Howard

**Affiliations:** 1 Department of Pharmacology, University of Cambridge, Cambridge, UK; 2 Wellcome-MRC Cambridge Stem Cell Institute, Cambridge, UK; 3 Department of Histology and Embryology, Faculty of Medicine, Masaryk University, Kamenice 3, 625 00 Brno, Czech Republic; 4 Laboratory of Genetics and Developmental Biology, Institut Curie, INSERM U934, CNRS UMR3215, Paris, France; 5 Sorbonne Université, Collège Doctoral, F-75005 Paris, France; 6 Department of Biomedicine, University of Basel, University Hospital Basel, Basel, Switzerland; 7 The Breast Cancer Now Toby Robins Research Centre, The Institute of Cancer Research, London, UK

**Keywords:** Mammary gland biology, Breast cancer, Breast development, Ductal carcinoma in situ, Organoids, Lobular breast cancer, Patient-derived xenografts, Resistance to endocrine therapy, In vivo live imaging, Lactation

## Abstract

The thirteenth annual workshop of the European Network for Breast Development and Cancer (ENBDC) Laboratories Annual Workshop took place on the 28–30 April 2022 in Weggis, Switzerland and focused on methods in mammary gland biology and breast cancer. Sixty scientists participated in the ENBDC annual workshop which had not been held in person since 2019 due to the global COVID-19 pandemic. Topics spanned the mammary gland biology field, ranging from lactation biology and embryonic development, single cell sequencing of the human breast, and stunning cutting-edge imaging of the mouse mammary gland and human breast as well as breast cancer research topics including invasive progression of the pre-invasive DCIS stage, metabolic determinants of endocrine therapy resistance, models for lobular breast cancer, and how mutational landscapes of normal breast during age and pregnancy determine cancer risk. The latest findings from participating researchers were presented through oral presentations and poster sessions and included plenty of unpublished work.

## Introduction

The European Network of Breast Development and Cancer (ENBDC) Laboratories Annual international workshop is specifically aimed at junior scientists, PhD students, postdoctoral fellows, and research associates to provide a forum to learn more about current research and methodology so that both informal and formal exchanges will advance their own research. For many of the ENBDC 2022 workshop participants, this was the first in person conference that they attended since the widespread cancellation of “live” conferences in early 2020 due to the COVID-19 pandemic which gave it a particularly animated atmosphere. Participants also included senior scientists who gave invited talks, as well as ENBDC committee members, who are leaders in breast biology and breast cancer research, providing abundant networking opportunities. A large amount of unpublished research was discussed during two lively poster sessions. The ENBDC workshop was started in 2009 and remains well-known for its welcoming spirit for newcomers in the field of mammary gland biology and breast cancer research. The aim of the ENBDC workshop is to raise awareness of each other’s research to foster contact, encourage technical exchange and collaboration, especially across disciplines, as well as to offer intellectual and information resources to the breast development and cancer research community worldwide.

## Meeting Report

The previous ENBDC Twelfth Annual Workshop held in 2021 was a virtual event [1] which allowed for more participants to join and whose success stoked the planning for the Thirteenth ENBDC Workshop which was organised without knowing whether it would be held virtually or in person. ENBDC has also started an online monthly progress report in the same spirit and with the same goals as its Weggis annual workshop. For those able to attend in 2022, the sun shined throughout the ENBDC Workshop, and we enjoyed lunch and coffee breaks outside where old- and new-comers met and mingled and were able to experience the magic of this wonderful meeting and place.

The meeting kicked off with a Keynote presentation by Louise Jones (Barts Cancer Institute, London, United Kingdom). Her presentation focused on her work around understanding the progression of ductal carcinoma in situ (DCIS) to invasive breast cancer. With the advent of screening, DCIS now represents 20% of all new cancer diagnoses but < 50% of cases will ever progress to invasive breast cancer so current management represents significant overtreatment. Understanding the mechanisms leading to progression would allow more tailored treatment. Tumours from DCIS are characterised by invasion of neoplastic cells through the myoepithelial cell barrier. However, the overall morphology of DCIS is heterogeneous with multi-clonal invasion [2]. Together, these features greatly limit the ability of clinicians and researchers to identify which DCIS will progress to invasive breast cancer, which remains an unmet clinical need. This led Jones to taking a closer look at the myoepithelial cells of DCIS and focusing on understanding the microenvironment of these tumours. Myoepithelial cells that express ανβ6 were found to promote tumour cell invasion through MMP9 and TGFβ- dependant mechanisms [3]. Further examination of factors influenced by ανβ6 found that fibronectin was upregulated, whereas, if fibronectin was knocked down, there was decrease in ανβ6 expression and tumour cells were inhibited from migration. From a microenvironmental perspective, DCIS tumours typically have a periductal necklace of new blood vessel formation. Jones’s work has focused further on understanding the relationship between ανβ6 and angiogenesis. Mouse models increasing ανβ6 expression found enhanced angiogenesis compared to ανβ6 negative mice and in vitro models demonstrated a direct effect of ανβ6 on vessel formation. In human tissues, it was observed that the ανβ6 + ducts had an increased diameter compared to ανβ6 negative ducts and that the myoepithelial cells appeared stretched. To examine the impact of stretch on the myoepithelial cells, they were cultured on flexible culture dishes, where a vacuum was applied to stretch the cells. When this occurred, there was an upregulation of ανβ6 and fibronectin. Together these presented findings demonstrated the central activity of myoepithelial cells, ανβ6, TGFβ and fibronectin signalling to promote angiogenesis and progress DCIS to invasion.

Following the Keynote talk, Renée van Amerongen (University of Amsterdam, Netherlands) chaired the first session on “New models for studying mammary gland development. The session opened with Biancastella Cereser (Imperial College, London, UK) who presented her work on understanding a simple question: “How normal is normal?” in the context of the human breast, with the aim to enlighten the association between breast cancer and pregnancy. Cereser sequenced the whole genome of a range of healthy breast tissues, carefully selecting samples with negative histories of oral contraception use, cancer, obesity or known BRCA mutations, and including a range of age and parities. Epithelium and stroma were separated by laser capture microdissection and the latter was used to exclude common mutations. Surprisingly, Cereser found that the healthy breast can carry a substantial number of mutations that drive cancer in other organs, such as PTEN, KRAS, TP53, NOTCH2 and BRAF, but without displaying any indication of cancer development, and that the number of mutations increases with age. On the other hand, pregnancy, a stage of breast development associated with enormous epithelial expansion and subsequent apoptosis, does not increase the number of mutations, but instead it acts as a selection pressure that enables expansion of some clones and disappearance of others. Interestingly, the stromal compartment showed the opposite findings, with an increased number of mutations during pregnancy and an expanded clonal size with age, a phenomenon that is not yet understood. Cereser proposed a hypothesis linking age and parity with pregnancy-associated breast cancer: Pregnancy increases clonal size, but as burden of mutations increases with age, only in older pregnant woman, the chance of detrimental clone expansion is increased with 35 years of age being the turning point. More detail is available in her recent preprint [4].

Adrian Ranga (KU Leuven, Belgium) brought a bottom-up approach to understanding epithelial self-organisation. Researchers in Ranga’s team, making mammary and other types of 3D cultures, avoid use of the most widely utilized extracellular matrix (ECM) Matrigel, that supports epithelial growth but is, in fact, a black box, composed of more than 1800 different proteins [5]. Ranga presented a tuneable 3D microenvironment, composed of a polymer system based on polyethylene glycol hydrogels. In this system, several matrix properties like biodegradability, stiffness, and ECM composition can be modulated. This creates a plethora of options, but this large number of possible combinations presents a need for automated arraying and image analysis to gain the most out of the experiments. As an example of a breast-related study, Ranga presented data from 3D cultures of MCF10A cells, whose polarity is influenced by numerous factors from soluble ligands to ECM stiffness. While this may not be surprising, the interesting finding was that degradability of the ECM is a key morphogenetic feature that does not favour any specific phenotype but instead increases the overall phenotypic heterogeneity [6]. As well as these tuneable physical properties of the ECM, Ranga provided a tour through futuristic approaches to involve mechanical forces into the 3D cultures like “actuoids”, organoids in stretchable environment [7] and “magnetoids”, organoids comprising magnetised cells that can be forced to migrate inside epithelium by applying magnetic forces [8].

The second session was chaired by Zuzana Koledova (Masaryk University, Brno, Czech Republic) and focused on metabolic reprogramming in breast cancer. The first talk of this session was given by Andrea Morandi (University of Florence, Italy) who discussed the effect of metabolic deregulation and reprogramming in breast cancer. In the case of postmenopausal estrogen receptor positive (ER+) breast cancer, some patients demonstrate a resistance to aromatase inhibitors, which are standard treatment for these cancers. This resistance is likely due to estrogen independent activation of the ER in these patients. Morandi’s laboratory uses a panel of isogenic long-term estrogen deprived (LTED) breast cancer cells to model this resistance and has found that central carbon and amino acid metabolism are deregulated and under the control of miR-155 and miR-23b-3p which have prognostic and predictive value in this subset of breast cancer [9]. Follow-up studies revealed that LTED cells had an increase in lipid deposition and highlighted a potential metabolic vulnerability of the modelled therapy-resistant cancer cells [10]. ER + breast cancer cells that had undergone LTED, or not, were also treated with TOFA (an acetyl-CoA carboxylase, ACC1, inhibitor) and LTED cells were more sensitive. It was found that LTED treated cells in the presence of TOFA had higher levels of cellular reactive oxygen species which was not associated with mitochondrial function but rather to the peroxisomal activity which is highly enhanced in LTED. Using ex vivo explants developed from ER + HER2- patient derived xenografts (PDXs) Morandi found that TOFA reduces the explants’ volume and the in vivo experiments using PDX models are on-going. Together these findings suggest that the metabolism of cells can react rapidly and that it is not the strongest cells that survive but rather the most adaptable.

Oona Paavoliainen, PhD student at the University of Turku (Finland), gave a short talk presentation “Morphometric analysis of human breast using light sheet microscopy”. Determining the 3D architecture of the female human breast is challenging as the branching patterns are quite different to those of mice. The strategy included obtaining human breast tissue, sectioning this into small tissue pieces (5–10 mm), screening for terminal ductal lobular units (TDLUs), tissue clearing these small sections, followed by using light sheet microscopy to image the TDLUs. With this technique, she was able to determine length, angle, and number of branches of TDLUs and found these data consistent between individual tissue donors. It was previously thought that there are four different types of branching present in the mammary gland, where type 1 represents nulliparous tissue and type 4 is a more intense branching that occurs during pregnancy. However, most analysed tissue, despite parity, was of type 2. Using these measurements, the research group was able to recapitulate the branching dynamics using an *in silico* model expanding on a previous mathematical model [11]. The tips will elongate and bifurcate and will stop if they hit an existing duct or a predetermined maximum volume. Based on these data, the group was able to 3D bioprint organoid shapes that more faithfully replicated the branching angles of TDLUs than might have been achieved otherwise, leading to a model that is more like what is observed in vivo.

The second short talk of this session was presented by Dina Hany from the University of Geneva (Switzerland). The focus of her research is to target purine biosynthesis as a novel approach to overcome tamoxifen resistance in breast cancer. Resistance to endocrine therapies, which either target production of estrogen or the ERα itself, remains a major challenge when seeking to treat patients with ER + breast cancers. With a genome-wide CRISPR/Cas9 screen, she identified an upregulation of an enzyme involved in the *de novo* biosynthesis of purines, PAICS, as a determinant of the response to tamoxifen, a widely used selective estrogen receptor modulator. By overexpressing PAICS, she found that cells could become independent of estrogen for growth and were no longer responsive to tamoxifen. This was found to be due to cAMP-activated protein kinase A activity being elevated [12]. If PAICS were targeted, it could allow resistant cells to become re-sensitised to tamoxifen. This work supports combinatorial therapies targeting both PAICS and ERα to overcome endocrine therapy resistance.

The second day of the workshop began with Session 3 of the meeting which examined new models for studying specific types of breast cancer and was chaired by ENBDC founder, Momo Bentires-Alj from University of Basel (Basel, Switzerland). This session was opened by a presentation by George Sflomos from the Brisken laboratory in EPFL (Lausanne, Switzerland) who discussed xenograft models of invasive lobular carcinoma (ILC). ILC cells express high levels of estrogen and progesterone receptors, proliferate slowly with constant invasive features, and have a unique pattern of metastatic colonization compared to ER + breast cancer of no special type (NST). By performing intraductal injections into the milk ducts of virgin female mice, with either ER + cells from patients with primary ILC [13] or SUM-44PE and MDA-MB-134-VI, ER + metastatic ILC cells [14], the laboratory could model ILC in vivo. Using the latter technique and injecting into the milk ducts with SUM-44PE or MDA-MB-134-VI cells, a heterogeneous growth pattern is observed, and the cells appear to reside and grow predominantly from the ductal termini, whereas the ducts do not contain many ILC cells. These features together with the cells following the metastatic pattern of ILC recapitulate the characteristics of lobular cells. Examining the cancer genome atlas (TCGA) patient dataset identified lysyl oxidase like 1 (LOXL1), a collagen modifying enzyme, as being highly expressed in lobular carcinomas. Using the ILC intraductal model, described above, he demonstrated that mice dosed with BAPN, a LOX inhibitor, exhibited disrupted extracellular structures and decreased ER signalling, which subsequently led to decreased invasion, growth and metastasis of the tumours and opened new therapeutic strategies for ILC [14].

Along a similar theme, Jos Jonkers from Netherlands Cancer Institute (Amsterdam, Netherlands) presented a different strategy to investigate ILC using genetically engineered mouse models (GEMMs). Performing intraductal injections of lentiviral vectors expressing Cre-recombinase in *Brca1*^*F/F*^;*Trp53*^*F/F*^ (B1P) female mice generated tumours with long latency, which were morphologically similar to another GEMM model for ILC that had been described using the whey acidic protein (*Wap*) gene promoter [15]. This kind of model can be modified to look at different oncogenic drivers and explore not only ILC but also DCIS and ER + breast cancers. Working with Colinda Scheele, Jonkers found by using whole-mount imaging of the early lesions from mouse intraductal (MIND)-PDX models of DCIS, there were two models of growth phenotypes. These were either replacement or expansive growth from the site of injection and appeared to be predictive of invasive phenotype. Exome analysis detected higher copy number variations in cells with higher invasive potential, whereas, recurrent aberrations were observed in *CCND1*, *MYC* and *FGFR1*. As a last point, Jonkers raised the question of what the best animal models are to examine different kinds of breast cancers. Currently he is developing a rat model to examine ER + breast cancer phenotypes and has thus far found more homogeneous tumours where a good incidence of DCIS progresses to invasive disease.

Colinda Scheele, a previous DeOme prize winner, and new group leader at from the VIB-KU Leuven Centre for Cancer Biology (Leuven, Belgium) presented her work on the multi-dimensional imaging of tumour initiation in the breast. Her laboratory has developed a technique to conduct longitudinal intravital microscopy using a mammary imaging window with a replaceable lid [16]. Using intraductal injections of TAT-Cre protein into *Brca1*^*F/F*^;*Trp53*^*F/F*^;*R26-Confetti* mice, it was found by looking at the whole gland, that only few *Brca1*^*−/−*^;*Trp53*^*−/−*^ confetti clones had transformed after > 200 days of lineage tracing. The vast majority of *Brca1*^*−/−*^;*Trp53*^*−/−*^ confetti clones spread over large areas of the mammary ducts, but didn’t change the morphology of the gland, nor did a cancer phenotype develop. Using intravital imaging, the Scheele laboratory examined the clone size distribution of clones in normal Confetti mice compared to *Brca1*^*F/F*^*/Trp53*^*F/F*^ Confetti mice. Through the estrous cycle, it was found that the same clones would rapidly expand and regress over time, where if the mice were ovariectomised the *Brca1*^*F/F*^*/Trp53*^*F/F*^ mice had only small clones, suggesting that the estrous cycle is a driver of field cancerization. Scheele took a modelling approach to understand what limits further expansion of cancerous field within the mammary gland, which revealed that the ductal geometry of the mammary gland led to spreading of mutant cells being restricted.

The first short talk of this session, “MYC promotes immune suppression in TNBC via inhibition of IFN signalling”, was presented by Dario Zimmerli, a postdoc in Jos Jonker’s laboratory (Netherlands Cancer Institute, Amsterdam, Netherlands). He compared the immune response of four triple negative breast cancer (TNBC) GEMMs that were BRCA1 proficient or deficient and either had normal or engineered overexpression of MYC: $$WapCre;Trp{53^{F/F}}\left( {WP} \right),$$$$WapCre;Trp{53^{F/F}};$$$$Col1a{1^{invCAG - Myc - IRES - Luc/ + }}\left( {WP - Myc} \right),$$$$WapCre;Brca{1^{F/F}};$$$$Trp{53^{F/F}}\left( {WB1P} \right)$$ and $$WapCre;Brca{1^{F/F}};Trp{53^{F/F}};$$$$Trp{53^{F/F}};$$$$Col1a{1^{invCAG - Myc - IRES - Luc/ + }}\left( {WB1P - Myc} \right)$$ [15, 17]. He found that in the models that had MYC overexpression, there was a dramatic decrease in lymphocyte infiltration into the tumours, along with immune signature loss. Generating mammary epithelial organoids with lymphocyte co-cultures revealed that MYC overexpression also inhibited recruitment and activation of the lymphocytes. Overexpression of MYC suppresses innate immunity, blocking induction of interferon signalling and tumour growth inhibition [17].

The final presenter of this session, Silke Chalmers (Aarhus University, Denmark) revealed strategies to uncover the calcium conversation between breast cancer and the brain microenvironment. Up to 30% of metastatic breast cancer patients develop lesions in the brain, where the metastatic cells adapt to thrive in a different microenvironment. To explore whether calcium (Ca^2+^) signalling contributes to the adaption of breast cancer to the brain microenvironment, Silke developed an in vitro brain metastases model through co-culturing human neural matrices comprised of astrocytes and neurons with MDA-MB-468 breast cancer cells. Distinct genetically encoded Ca^2+^ sensors were expressed in each cell population. Using high-content imaging and single cell analysis, identified a spatial relationship between Ca^2+^ signalling events in breast cancer and brain cells, providing evidence that breast cancer cells experiencing cellular stresses can communicate via a Ca^2+^ signal to neighbouring neural cells. This work suggests that Ca^2+^ communication may play a role in breast cancer adaptation and survival in brain microenvironments.

The student/postdoc session chairs of this year were Jakub Sumbal, a PhD student from the Masaryk University, Brno, Czech Republic and Institut Curie, Paris, France and Alecia-Jane Twigger, a postdoctoral researcher, from Cambridge University, Cambridge, UK. They chose the theme of the session, Lactation, as well as the speakers for their session. This session began with Felicity Davis (Aarhus University, Aarhus, Denmark & EMBL Australia, Sydney, Australia) who brightened up the last conference day with numerous fluorescent movies of contracting lactating mammary glands. During lactation, mammary myoepithelial cells differentiate into highly contractile elements that respond to oxytocin elevation by ejecting milk out of the gland to support the newborn’s nutrition; this phenomenon is well-known, however, it is not often you can directly see it. But that is what the Davis team do, by utilising volumetric live-imaging of ex vivo lactating mammary gland fragments from genetically-engineered mice that expresses a fast calcium sensor (GCaMP6f). In the movies Davis presented, it is evident, that after oxytocin stimulation, Ca^2+^ signalling in myoepithelial cells precedes the contraction of mammary alveoli. Moreover, she observed that the calcium signal is capable of travelling through the ductal and alveolar network of the lactating gland in an organized manner, suggesting a cell-cell Ca^2+^ signal transduction. And indeed, by high-resolution 3D imaging of mammary alveoli, her team found that cytoplasms of star-shaped, fully differentiated myoepithelial cells are interconnected and confirmed the presence of a gap junction protein Cx43 at the myoepithelial cell-cell contact. Davis’s work thus leads to a hypothesis that it is the intracellular Ca^2+^ signalling that orchestrates proper myoepithelial contraction to ensure the most efficient milk ejection [18].

Geula Hanin (University of Cambridge, Cambridge, UK) began her talk with a reminder that epigenetic changes underline mammary adaptation for subsequent pregnancy [19] and that many genes in mammary gland are parentally imprinted. She followed with a presentation of recent data on developmental defects of pups in a mouse model that lacks the control of parental imprinting. Those pups become obese and grow faster than control pups, due to upregulated milk protein synthesis in mice that lack the imprinting regulation. Finally, Hanin hypothesised, that the mammary gland, the source of postnatal nutrition, is similar to the placenta, the prenatal nutrition source, where both have a high rate of parentally imprinted genes [20].

In the first short talk of this session, Aurélie Chiche (Institut Pasteur, Paris, France) presented her work on cellular senescence during mammary gland development. Cellular senescence is difficult to study, especially in vivo, due to the lack of reliable markers. However, Chiche found by using a combination of senescence markers, senescence-associated beta-galactosidase staining, as well as expression of p16, p19 and p21 detected in luminal epithelium during mammary gland involution, there is a developmental stage-specific activation of the senescence program. For functional studies, she used germline p16 and p21 knock-out mouse model (“senescence-free mouse”) and demonstrated that the p16/p21 axis is important for proper mammary development as knock-out mice experienced defects in mammary alveolar development, increased epithelial proliferation with insufficient pup feeding abilities. To provide epithelial specificity, Chiche used a set of in vitro organoid experiments [21] to show that p16/p21 knockouts displayed defects in mammary morphogenesis and milk production. This suggests that p16/p21 axis is important for lactational differentiation of the mammary gland.

The last short talk of the conference was presented by Elena García-Trevijano (Universidad de Valencia, Valencia, Spain) who spoke about a protease, calpain 2 (CAPN2), and its functional switch between normal and neoplastic development. During lactation CAPN2 is associated with E-Cadherin on epithelial junctions, putatively clearing cell-cell connections to enable cell shedding into the lumen, however it becomes nuclear localized in breast cancer cells [22]. CAPN2 mediates, via interaction with LIM domain kinase 1, phosphorylation of nuclear cofilin 1 that is necessary for proper nuclear dynamics. Deletion of CAPN2 in breast cancer cells leads to lack of cofilin phosphorylation, mitotic defects, and multinucleated cells. Taken together, this data suggests a breast cancer-specific subcellular distribution and role of CAPN2 [23].

The Best Short talk/The DeOme Award was presented to Oona Paavolainen, a doctoral student with Emilia Peuhu (Institute of Biomedicine at University of Turku) where her project focuses on mechanobiological regulators of mammary gland development. Poster Prizes were awarded to Vera Van der Noord from Bob van de Water’s lab at Leiden University, Leiden, The Netherlands for her poster “Tyrosine kinase inhibitors sensitize triple-negative breast cancer to CDK12/13 inhibition by blocking ABCG2 drug efflux”, Lisa Hess from Thomas Reinhecke’s lab at Albert-Ludwigs-University of Freiburg, Freiburg, Germany for her poster “Stage-dependent functions of dipeptidyl peptidase 9 in mammary cancer progression” and Claudia Carabana from Silvia Fre’s lab at the Institut Curie, Paris, France for her poster “Defining cell fate specification of mouse mammary stem cells in 4D”.

We also had the privilege of seeing Marie-Ange Deugnier, prior to her retirement from the Institut Curie, Paris. Marie-Ange has been a familiar face at the ENBDC annual workshop, where her scientific expertise, thoughtful contributions to discussions, and presence on the disco dancefloor will be missed. The participants decided that the 2023 Annual Workshop will be preceded by a one-day workshop just for PhD students (who will plan the programme for this “Students-only Day” ENBDC Workshop to be held on the 27th of April 2023. The main ENBDC workshop will occur from 28th to 29th of April 2023 and will chaired by Walid Khaled from Cambridge University, Cambridge, UK and co-chaired by Silvia Fre from Institut Curie, Paris, France. The postdoctoral chair will be Silke Chalmers from Aarhus University, Denmark and the PhD chair will be Tanne van der Wal from University of Amsterdam, Netherlands. This new development of the ENBDC annual workshop format bodes well for its future as it can only strengthen its ability to bring together an international group or early researchers with diverse research interests who are keen to network and learn about the vast methodologies that are available to study this fascinating organ and terrible disease.

## Declarations

Ethics MB-A, and BAH are Editorial board members of the Journal of Mammary Gland Biology and Neoplasia.
